# Strain-Modulated Electronic Structure and Infrared Light Adsorption in Palladium Diselenide Monolayer

**DOI:** 10.1038/srep39995

**Published:** 2017-01-04

**Authors:** Xiaobiao Liu, Hongcai Zhou, Bo Yang, Yuanyuan Qu, Mingwen Zhao

**Affiliations:** 1 School of Physics & State Key Laboratory of Crystal Materials, Shandong University, Jinan, Shandong, 250100, China

## Abstract

Two-dimensional (2D) transition-metal dichalcogenides (TMDs) exhibit intriguing properties for both fundamental research and potential application in fields ranging from electronic devices to catalysis. Based on first-principles calculations, we proposed a stable form of palladium diselenide (PdSe_2_) monolayer that can be synthesized by selenizing Pd(111) surface. It has a moderate band gap of about 1.10 eV, a small in-plane stiffness, and electron mobility larger than that of monolayer black phosphorus by more than one order. Additionally, tensile strain can modulate the band gap of PdSe_2_ monolayer and consequently enhance the infrared light adsorption ability. These interesting properties are quite promising for application in electronic and optoelectronic devices.

Two-dimensional (2D) transition-metal dichalcogenides (TMDs) represent a new family of 2D materials beyond graphene. They have a general formula of MX_2_ where M is a transition metal from group 4–10 and X is a chalcogen (S, Se, or Te). In contrast to graphene and their bulk materials, some 2D-TMDs are semiconducting with a sizable band gap, partially due to quantum-confinement effects. So far, theoretical and experimental efforts have been mainly focused on semiconducting 2D-TMDs with M = Mo and W^1^,^2^,^3^,^4^, due to potential applications in terahertz switch^5^, lasers and LEDs^6^, ambipolar ionic liquid gated field-effect transistor^7^, photo detectors^1^, and so on^8^. In a recent work^9^, PtSe_2_ monolayer was synthesized via a single step of direct selenization of a Pt(111) substrate and demonstrated to have potential application as valleytronics and photocatalyst.

Beside platinum, palladium (Pd) can also form binary compounds with Se with different stoichiometries and crystal structures, such as trigonal α-Pd_4_Se, tetragonal PdSe, cubic Pd_17_Se_15_, monoclinic Pd_7_Se_2_, and orthorhombic PdSe_2_^10^. Under ambient conditions, PdSe_2_ crystal appears in an orthorhombic structure with a space group of Pbca similar to the case of PdS_2_^11^,^12^. Using first-principles calculations, Sun *et al*., proposed a PdSe_2_ monolayer with semiconducting nature and high Seebeck coefficients^11^. So far, PdSe_2_ monolayer has not been achieved experimentally, partially due to the strong layer binding energy ~190 meV/atom in orthorhombic PdSe_2_ bulk crystal which makes the exfoliation of PdSe_2_ monolayer difficult. However, the recently success in the synthesis of PtSe_2_ monolayer on Pt(111) substrate via selenization opens an avenue towards this goal.

In this manuscript, we proposed a novel stable configuration of PdSe_2_ monolayer that can be synthesized by selenizing Pd(111) surface, as shown in Fig. 1(a). Our first-principles calculations indicate that it is energetically comparable to the Pbca phase^11^. Phonon spectrum analysis confirms its dynamics stability. This PdSe_2_ monolayer has an indirect-band-gap of about 1.10 eV, suitable for application in infrared light region. It also has high electron mobility of about 9800–42000 cm^2^v^−1^s^−1^ which is larger than that in monolayer black phosphorus by more than one order. The in-plane stiffness of 54 J/m^2^ is only half of MoS_2_, allowing a large tensile strain up to 35%. Additionally, tensile strain can significantly reduce the band gap and consequently enhance the infrared light adsorption ability. These interesting properties are quite promising for application in electronic and optoelectronic devices.

## Results and Discussion

### Atomic structure and stability

**Figure 1:**
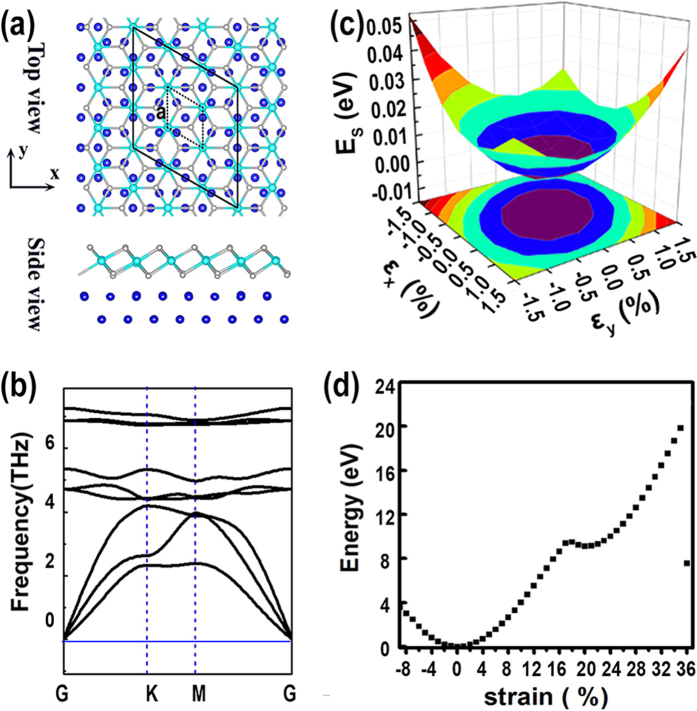
(**a**) The top view and side view of atomic configuration for PdSe_2_ monolayer on Pd(111) surface. The blue and cyan balls are Pd atoms and gray ball are Se atoms. The small and large black parallelograms represent primitive lattice for monolayer PdSe_2_ and PdSe_2_-Pd(111) superstructure, respectively. The black arrows indicate the x- and y-direction for top view. (**b**) The phonon spectrum of PdSe_2_ monolayer. (**c**) Three-dimensional plot of strain energy *E*_*S*_ (see text) depending on strain of *ε*_*x*_ and *ε*_*y*_(see text). The color filled contour of the fitted formula is also plotted. (**d**) The energy evolution of PdSe_2_ monolayer under biaxial compressive and tensile strains. The energy at the equilibrium state was set to zero.

The atomic structure of a freestanding PdSe_2_ monolayer with a P-3M1 space group is similar with that of PdSe_2_ monolayer grown on the Pd(111) surface, as shown in Fig. 1(a). Each Pd atom bonds to six Se atoms and each Se atom is coordinated by three Pd atoms. All the Pd atoms are on a same plane sandwiched by two Se planes. The thickness of the freestanding PdSe_2_ monolayer measured from the distance between the two Se planes is 2.631 Å. The Pd-Se bond length is about 2.528 Å. The optimized lattice constant of the hexagonal lattice is 3.739 Å, quite close to that of the PtSe_2_ monolayer, 3.7 Å 9. Notably, a 3 × 3 PdSe_2_ monolayer (11.217 Å) matches well with the 4 × 4 Pd(111) surface (11.186 Å), which implies high plausibility of growing PdSe_2_ monolayer on Pd(111) surface via selenization. This allows us to build a PdSe_2_/Pd heterostructure by placing a 3 × 3 PdSe_2_ on Pd(111) surface without considering the small lattice mismatch (~0.3%). Compared with freestanding monolayer, the buckling of the supported PdSe_2_ increases to 2.710 Å and the bond length vary from 2.51 Å to 2.61 Å with an average of 2.55 Å, due to the substrate effect. The distance between PdSe_2_ monolayer and substrate is about 2.16 Å, and the binding energy between them is −1.04 eV per PdSe_2_ unit. The PdSe_2_-Pd(111) interaction is much stronger than the van der Waals (VDW) interaction in graphite (~−36 meV/atom), making the exfoliation of PdSe_2_ from the Pd substrate difficult.

It is noteworthy that the lattice structure of hexagonal PtSe_2_ monolayer differs significantly from that cut from orthorhombic PdSe_2_ crystal^11^. The latter case has orthorhombic symmetry with four-coordinated Pt atoms and two-coordinated Se atoms, and is slightly stable than the hexagonal structure by about 26 meV/atom. However, the hexagonal PdSe_2_ monolayer matches well with the Pd(111) surface in both symmetry and lattice constant along with a negative binding energy (−1.04 eV per PdSe_2_ unit) and thus has high plausibility on the Pd(111) surface as the surface is selenized^9^.

To confirm the dynamic stability of the PdSe_2_ monolayer, we calculated the phonon spectrum using a finite displacement method implemented the Phonopy code interfaced the VASP code^13^,^14^. It was found that the phonon spectrum is free from imaginary frequency modes, as shown in Fig. 1(b), suggesting the dynamic stability of the PdSe_2_ monolayer. Additionally, there is a small energy gap between the acoustic and optical branches in the phonon spectrum. Such an energy gap can protect the vibration of acoustic modes from being interrupted by optical phonon^15^,^16^,^17^, which leads to the resonators with higher quality factor than the graphene resonators^18^.

### Mechanical properties

Strain is not evitable as a monolayer is grown on a substrate. We therefore investigated the mechanical properties PdSe_2_ monolayer from first-principles. To obtain the elastic modulus in harmonic range, we employed a rectangular supercell and applied strains along x- and y-direction, as shown in Fig. 1(a). The energy (*E*_*s*_) dependence of strains is illustrated in Fig. 1(c). The data is fitted to a two-dimensional polynomial expressed by 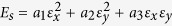, where *ε*_*x*_ and *ε*_*y*_ represent the strains applied along x- and y-direction, respectively. The total energy of system at the equilibrium state was set to zero. It was found that the two parameters a_1_ and a_2_ are almost identical due to the isotropy in the honeycomb symmetry^19^. The poisson’s ratio (*ν*) and in-plane stiffness (*C*) can be obtained as 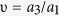 and 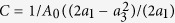
20, where A_0_ is the equilibrium area of the structure. The calculated poisson’s ratio (0.29) is close to those of MoS_2_ (0.25) and silicene (0.30) but larger than that of graphene (0.16). The in-plane stiffness of PdSe_2_ monolayer, 54 J/m^2^, is much smaller than those of MoS_2_ (123 J/m^2^) and graphene (335 J/m^2^)^19^,^21^, suggesting that PdSe_2_ monolayer is softer than both graphene and MoS_2_ monolayer.

The softness of PdSe_2_ monolayer was also confirmed by the energetic and structural evolution as a function of biaxial strain. Figure 1(d) gives the energy increase of PdSe_2_ monolayer as the biaxial strain varies from −9% (compression) up to 35%. It is interesting to see that energy varies continuously without abrupt decrease, indicating that no Pd-Se bonds are broken in this region. As the tensile strain exceeds 35%, energy decreases drastically and irreversible structure modification along with Pd-Se bond breakage takes place. The two energy local minima at ε = 0 and 20.4% correspond to the equilibrium state and a metal-stable state of PdSe_2_ monolayer. The later configuration has the Pd-Se bond length of 2.639 Å, slightly longer than that of the equilibrium state by about 4.4%, but the thickness is greatly reduced to 0.918 Å compared to the equilibrium state value 2.631 Å. The metastable state represents a low-buckled configuration of PdSe_2_ monolayer under high tensile strain. Similar results have also been reported for silicene, but the critical tensile strain in PdSe_2_ monolayer is much larger than that in silicene^21^.

### Electronic properties

**Figure 2:**
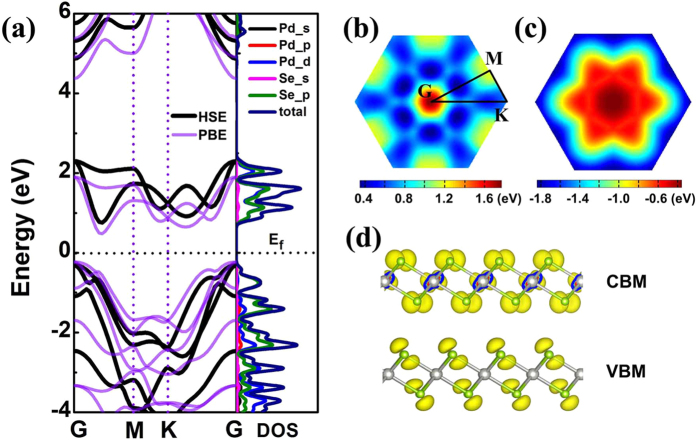
(**a**) Bands structures of PdSe_2_ monolayer along the high symmetric points in the BZ (right) and orbital-resolved electron density of states (left). The energy at the Fermi level is set to zero. The black lines and purple lines represent the data obtained from HSE and PBE functionals, respectively. M (1/2, 0, 0), G (0, 0, 0) and K (1/3, 1/3, 0) represent high symmetric points in the reciprocal space. The momentum-dependent energy distribution in BZ for (**b**) the lowest unoccupied band and (**c**) the highest occupied band. (**d**) Electron density profiles of the Kohn-Sham wavefunctions for CBM and VBM.

We then calculated the electronic structure of PdSe_2_ monolayer from first-principles. We employed the PBE functional and Heyd–Scuseria–Ernzerhof (HSE) screened Coulomb hybrid density functional^22^ in the framework of DFT. The band structure and electronic density of states (DOS) near the Fermi level are illustrated in Fig. 2(a). Both functionals gave the semiconducting nature of PdSe_2_ monolayer with an indirect band gap, similar to the case of orthorhombic PdSe_2_^11^. The valence band maximum (VBM) resides at the Г (0, 0, 0) point while the conduction band minimum (CBM) locates in between Г (0, 0, 0) and M (0.5, 0, 0). Six valleys are therefore found in the whole Brillouin zone as shown in Fig. 2(b), which gives opportunities to valley devices. The PBE functional underestimated the band gap value (0.7 eV) compared with HSE functional (1.10 eV). The band gap of PdSe_2_ monolayer is slightly smaller than that of PtSe_2_ monolayer (1.2 eV)^9^ and orthorhombic PdSe_2_(1.43 eV)^11^ and suitable for adsorbing infrared light. From the orbital-resolved electron density of states shown in Fig. 2(a), we can see that the VBM is mainly contributed by the 4p orbits of Se atoms, while the CBM comes mainly from the 4d orbits of Pd and 4p orbits of Se. This is consistent with the features of the Kohn-Sham wavefunctions of the VBM and CBM plotted in Fig. 2(d). Considering Pd is a heave element, we also took the spin-orbit coupling (SOC) effect into account in electronic structure calculations. It was found that the energy degeneracy at some highly-symmetric points in BZ is lifted due to SOC (Fig. S2 in Supplementary Information). Both the valence and conduction bands are shifted downward and the global indirect band gap is decreased by about 0.2 eV. The reduced band gap would lead to red shift of optical adsorption peaks and thus facilitate the adsorption of infrared light.

When PdSe_2_ monolayer is grown on Pd(111) substrate, the energy bands of semiconducting PdSe_2_ and metallic Pd substrate mix together. Slight electron redistribution takes place in PdSe_2_ monolayer. About 0.05 electrons per Se atom transfer from upper Se layer to down Se layer, while the electron transfer from PdSe_2_ monolayer to substrate is almost negligible (~0.007 |e| per PdSe_2_ unit). This slightly reduces the workfunction of PdSe_2_ to 5.20 eV compared with that of freestanding PdSe_2_ monolayer (5.40 eV). We also calculated the electronic structures of PdSe_2_ multilayers built by stacking PdSe_2_ monolayers with different patterns. For the PdSe_2_ bilayer, AA stacking pattern is energetically more favorable than AB pattern (Supplementary Information). The interlayer interaction greatly reduces the band gap of 2D PdSe_2_. The band gap of the PdSe_2_ bilayer with AB pattern is only 0.33 eV (PBE), while the energetically preferred AA pattern becomes metallic.

We also calculated the electron mobility of PdSe_2_ monolayer using the phonon-limited scattering model^23^,^24^. In the method, the electron mobility can be evaluated using the expression^24^,^25^,^26^, 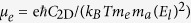; where *e* is the electron charge, *ℏ* is Planck’s constant divided by 2π, *k*_*B*_ is Boltzmann’s constant and *T* is the temperature. *m*_*e*_ is effective mass of electron in the transport direction (either m_x_ or m_y_ along the x and y direction, respectively) and *m*_*a*_ is the averaged effective mass of that along x and y direction determined by 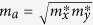. Deformation potential constant of CBM (*E*_*l*_) for electrons along the transport direction is defined by 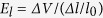, *ΔV* is the energy change of CBM, *l*_0_ is the lattice constant in the transport direction and Δ*l* is the change of *l*_*0*_. The elastic modulus *C*_*2D*_ of the longitudinal strain in the propagation directions of the longitudinal acoustic wave is derived from 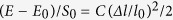; where *E* is the total energy and *S*_*0*_ is the lattice area at equilibrium for 2D system. The electron mobilities along x- and y-direction are 9800 cm^2^v^−1^s^−1^ and 42000 cm^2^v^−1^s^−1^ at 300 K, respectively. These values are much higher than that in monolayer black phosphorus by more than one order of magnitudes. Such high electron mobility is quite promising for improving the photocatalytic activity of PdSe_2_ monolayer, because the photo-generated electrons can be easily transferred^9^.

**Figure 3:**
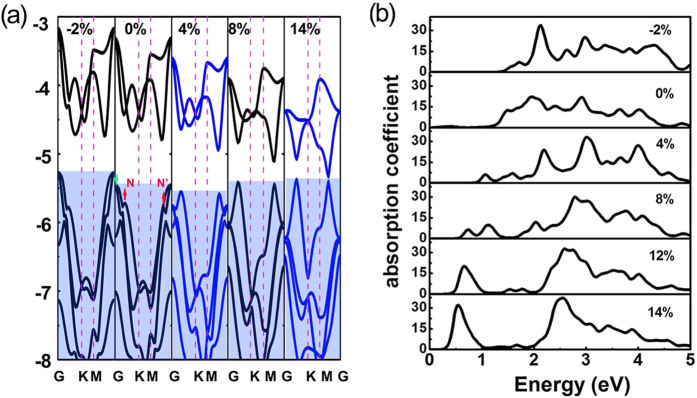
(**a**) The electronic band structures of PdSe_2_ monolayer under different strains obtained from PBE functional. The vacuum level is set to 0 eV. The regions of valence bands are covered by translucent blue. (**b**) Strain-dependent absorption coefficient of PdSe_2_ monolayer calculated by using HSE functional. The insert number donates the biaxial strain.

The electronic structure modification of PdSe_2_ monolayer in response to tensile strain was then investigated. With the increase of tensile strain, the band gap of PdSe_2_ monolayer decreases and comes to close as the tensile is larger than 14%. More interestingly, compressive strain also reduces the band gap of PdSe_2_ monolayer. To reveal the origins of the strain-induced band gap modulation, we plotted the band structures of PdSe_2_ monolayer under difference strains in Fig. 3(a). From this figure, we can see that under a tensile strain, the CBM move downwards while the VBM varies slightly, reducing the band gap. This may be related to the enhancement of the Se_4p and Pd_4d interaction due to the shorten distance between the Se plane and Pd plane. Under a compressive strain, however, the VBM is sensitive to strain and shifts upwards, leading to band gap reduction. Besides, it is interesting to see that tensile strain decreases the energy of the G points (illustrated by green arrow in Fig. 3(a)), and lifts the energies of the N and N’ points (illustrated by red arrow in Fig. 3(a)). As the tensile strain is larger than 4%, the energy difference between the N and N’ points becomes very small, making the PdSe_2_ a quasi-direct band semiconductor.

### Optical adsorption

The strain-induced band gap reduction affects the optoelectronic properties of PdSe_2_ monolayer. We calculated the imaginary part of complex dielectric function using the expression:


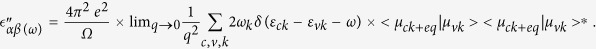


The indices *c* and *v* refer to conduction and valence band states respectively. *u*_*c**k***_ is the cell periodic part of the orbitals at the k-point **k.** *ω* is the frequency of the incident photon. The real part of the dielectric function 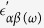 is obtained from the imaginary part with the Kramers-Kroning relations. The optical absorption coefficient is given by 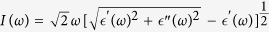; where 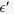
and 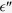 is the real and imaginary part of complex dielectric function. The optical absorption coefficient I(ω) calculated using HSE functional is plotted in Fig. 3(b). At equilibrium state, the adsorption peaks locate in the visible light and ultraviolet light region. A small compression has little affect on the adsorption properties. When a tensile strain is applied to PdSe_2_ monolayer, adsorption peaks come to appear in the low energy region, due to the band gap reduction. Meanwhile, the adsorption to visible light is partially screened. For example, under the tensile strains larger than 12%, the adsorption coefficients are almost zero in the energy region from 1.0–2.0 eV. These fascinating properties are quite promising for infrared detectors where the screening of visible light adsorption is needed.

## Conclusion

In summary, based on first-principles calculations, we proposed a stable form of palladium diselenide (PdSe_2_) monolayer that may be synthesized by selenizing Pd(111) surface. The PdSe_2_ monolayer possesses a moderate band gap of about 1.10 eV and high electron motilities of about 9800–42000 cm^2^v^−1^s^−1^, suitable for the electronic and optoelectronic devices working at infrared light region. The band gap of PdSe_2_ monolayer can be modulated by applying tensile strains. With the increase of tensile strain, the band gap decreases and comes to close as the tensile strain is larger than 14%. Consequently, the light adsorption ability of the PdSe_2_ monolayer in infrared light region is greatly enhanced. These findings are quite promising for application in electronic and optoelectronic devices.

## Methods

Our first-principles calculations were performed within density-functional theory (DFT) using the Vienna ab initio simulation package known as the VASP code^27^,^28^,^29^. The projector augmented wave method (PAW)^30^,^31^ was used to describe the electronic-ion interaction. The energy cutoff of the plane waves was set to 450 eV with an energy precision of 10^−5^ eV. The electron exchange–correlation function was treated using a generalized gradient approximation (GGA) in the form proposed by Perdew, Burke, and Ernzerhof (PBE)^32^. The Monkhorst-Pack *k*-point meshes^33^ for the Brillouin zone (BZ) sampling used in structural optimization and electronic structure calculations are 8 × 8 × 1 and 15 × 15 × 1, respectively. The primitive cell contains one Pd atom and two Se atoms which is periodically repeated along the x- and y-directions. A vacuum region up to 15 Å was applied along the z-direction to exclude the interaction between adjacent images. Both atomic positions and lattice vectors were fully optimized using the conjugate gradient (CG) algorithm until the maximum atomic forces were less than 0.0005 eV/Å. The lattice constants of the orthorhombic PdSe_2_ crystal (a = 5.752 Å, b = 5.926 Å) obtained from our calculations agree well with the experimental data (a = 5.746 Å, b = 5.868 Å)^12^, confirming the validity of this strategy.

## Additional Information

**How to cite this article**: Liu, X. *et al*. Strain-Modulated Electronic Structure and Infrared Light Adsorption in Palladium Diselenide Monolayer. *Sci. Rep.* **7**, 39995; doi: 10.1038/srep39995 (2017).

**Publisher's note:** Springer Nature remains neutral with regard to jurisdictional claims in published maps and institutional affiliations.

## Supplementary Material

Supplementary Information
