# Supplementary material for: Strain-Modulated Electronic Structure and Infrared Light Adsorption in Palladium Diselenide Monolayer
Source: Sci Rep. 2017 Jan 4;7:39995. doi: 10.1038/srep39995 (PMC5209744; doi:10.1038/srep39995)
Supplement: Supplementary Information [file srep39995-s1.doc]

Supplementary Information for

Strain-Modulated Electronic Structure and Infrared Light Adsorption in Palladium Diselenide Monolayer

Xiaobiao Liu, Hongcai Zhou, Bo Yang, Yuanyuan Qu & Mingwen Zhao*

*School of Physics & State Key Laboratory of Crystal Materials, Shandong University, Jinan, Shandong, 250100, China.*


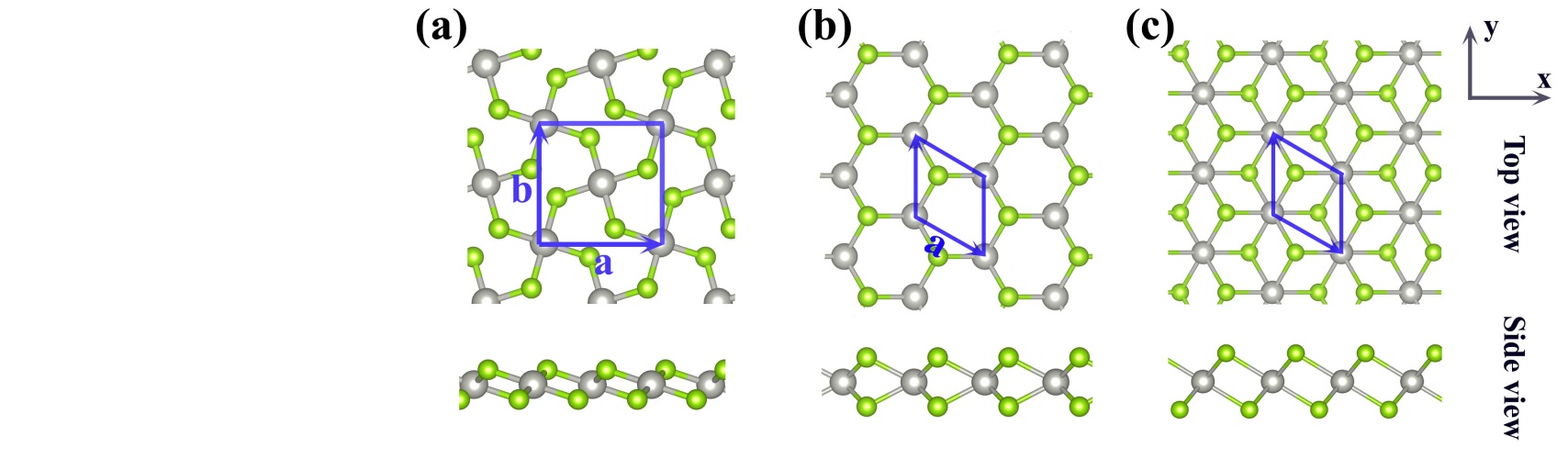


Fig. S1 The top and side views of three types of PdSe_2_ monolayers. (a) Orthorhombic PdSe_2_ (donated as o-PdSe_2_). (b) and (c) hexagonal PdSe_2_ monolayers (denoted as h-PdSe_2_-I and h-PdSe_2_-II, respectively) The green and gray atoms are for Se and Pd atom, respectively. The blue parallelograms represent primitive lattice.

Table S1 Energies (E) and structural parameters of PdSe_2_ monolayers with different configurations indexed in Fig. S1. BL represent represents the average Pd-Se bond length. The a^exp^ and b^exp^ are experimental lattice constants of bulk PdSe_2_ crystal.

|  | o-PdSe_2_ | h-PdSe_2_-I | | h-PdSe_2_-II |
| --- | --- | --- | --- | --- |
| E (meV/atom) | 0 | | 253 | 26 |
| Lattice constants (Å) | a=5.752; b=5.926  a^exp^=5.7457; b^exp^=5.8679 | | a=4.008 | a =3.739 |
| BL (Å) | 2.462 | | 2.605 | 2.527 |


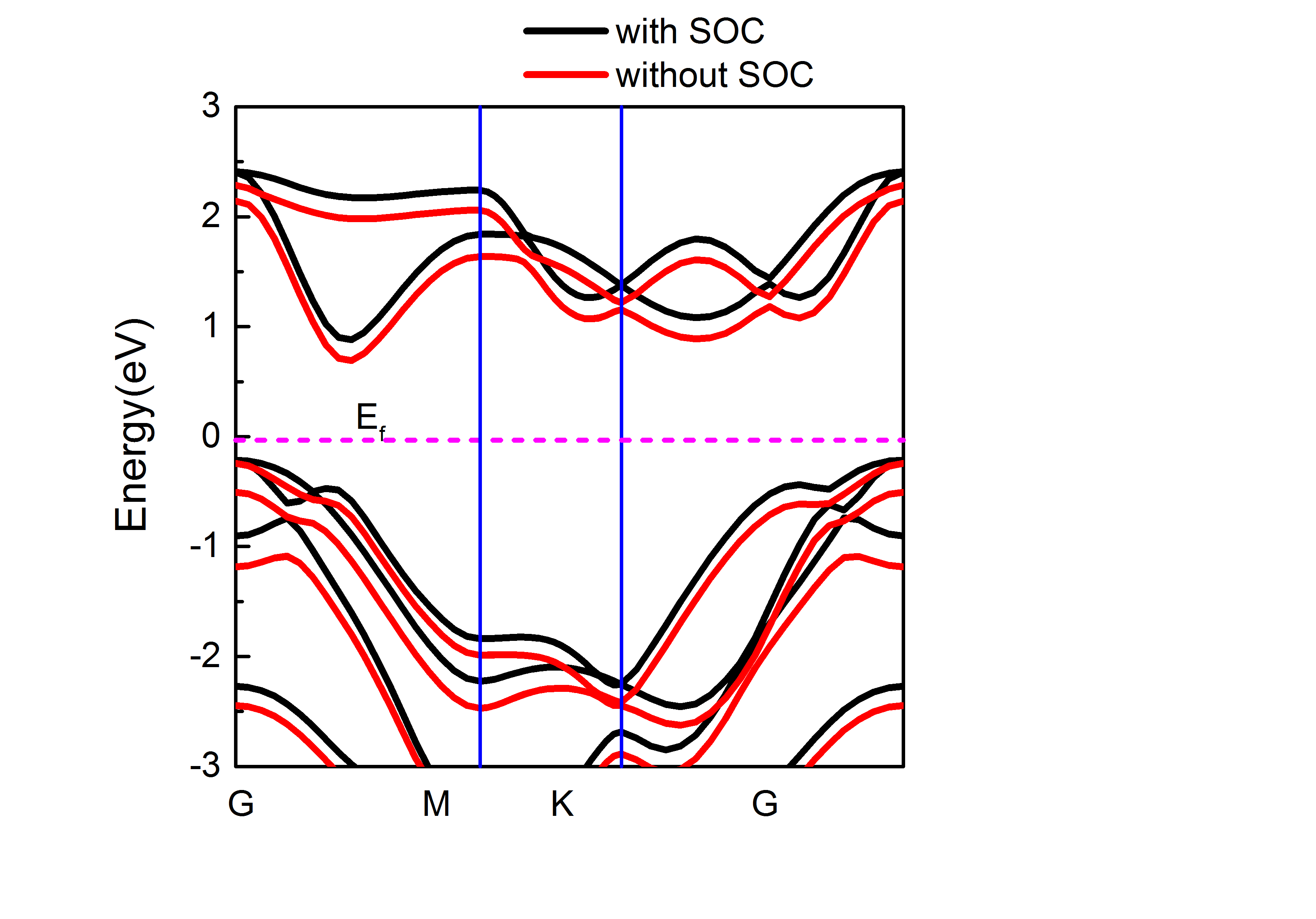


Fig. S2 The bands structure of PdSe_2_ monolayer calculated by HSE functional with and without SOC, M (1/2, 0, 0), G (0, 0, 0) and K (1/3, 1/3, 0) represent the high symmetric points in the reciprocal space. Fermi level is set to 0 eV.


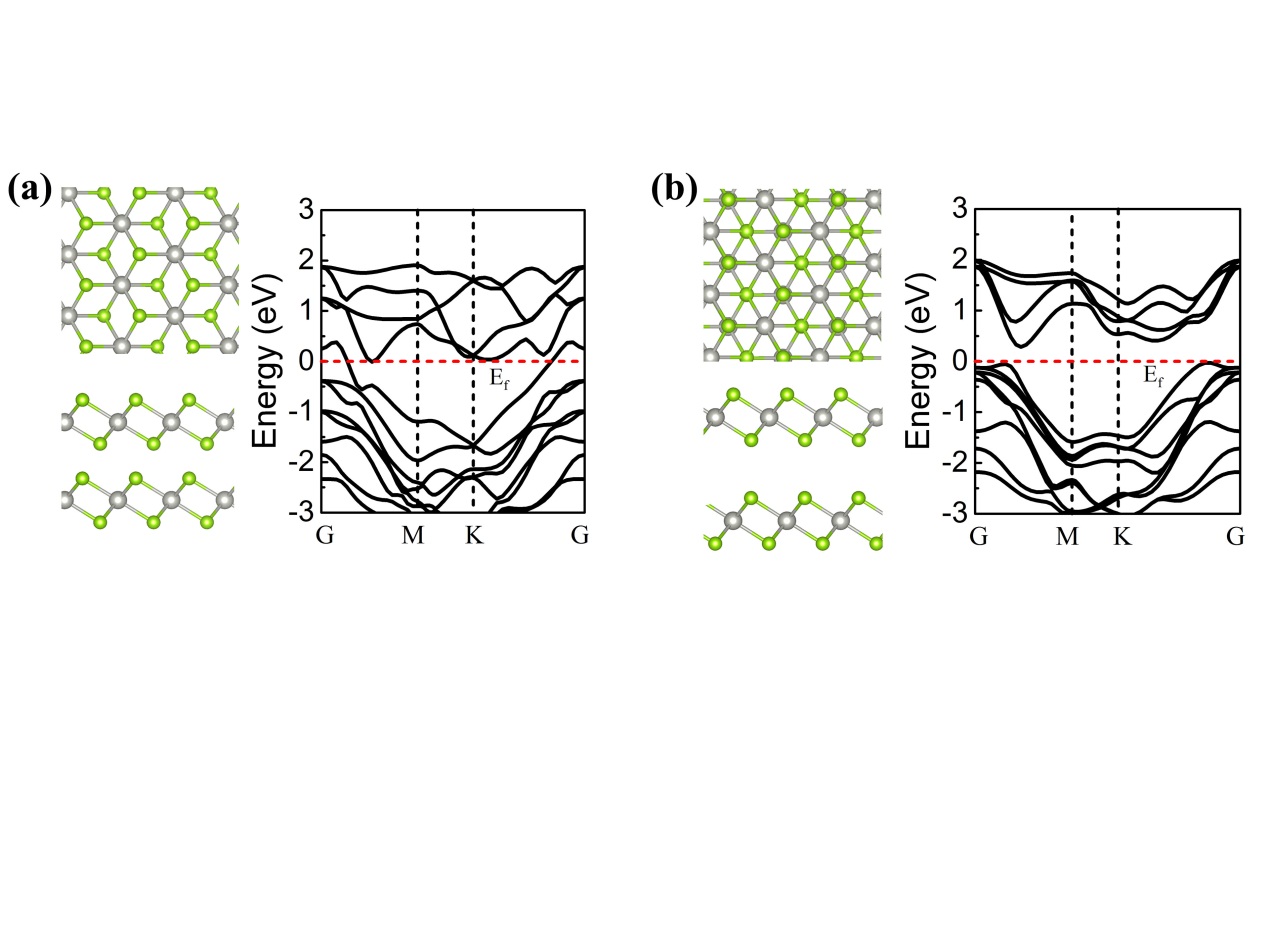


Fig. S3 Structure configure (left) and electronic band structures (right) of PdSe_2_ bilayers with (a) AA and (b) AB stacking patterns calculated by using PBE functional. The energy at the Fermi level is set to zero.

**References**

[1] Soulard, C. *et al.* Experimental and Theoretical Investigation on the Relative Stability of the PdS2- and Pyrite-Type Structures of PdSe_2_. *Inorg. Chem.* **43**, 1943-1949 (2004).
